# Exploratory axis factoring for identifying the self-esteem latent factors and their correlation with the life quality of persons suffering from vitiligo

**DOI:** 10.3389/fpsyg.2023.1200713

**Published:** 2023-12-15

**Authors:** Laszlo Fekete, Laszlo Barna Iantovics, Gyula Laszlo Fekete

**Affiliations:** ^1^Doctoral School, George Emil Palade University of Medicine, Pharmacy, Science and Technology of Targu Mures, Targu Mures, Romania; ^2^Department of Electrical Engineering and Information Technology, George Emil Palade University of Medicine, Pharmacy, Science and Technology of Targu Mures, Targu Mures, Romania; ^3^Department of Dermatology, George Emil Palade University of Medicine, Pharmacy, Science and Technology of Targu Mures, Targu Mures, Romania

**Keywords:** quality of life, self-esteem of vitiligo patients, self-esteem latent factors, life quality of vitiligo patients, psychometric properties of a questionnaire, the burden of vitiligo

## Abstract

**Objective:**

Our research aimed to measure the importance of self-esteem in assessing the disease burden in patients with vitiligo, which, according to our knowledge, had not been studied earlier. The purpose of this research study was to expand the state of knowledge regarding the influence of vitiligo on the quality of patients’ life, with a particular focus on their self-esteem. We have formulated the following two hypotheses which include H1: two latent factors characterize the self-esteem of patients with vitiligo; H2: the self-esteem of the patients with Vitiligo is correlated with their life quality, by influencing it to a high degree.

**Methods:**

We have used two validated questionnaires called Rosenberg (Q1), for the evaluation of self-esteem (for proving H1), and Dermatology Life Quality Index (DLQI) (Q2), to measure the health-related quality of life of patients (for proving H2). Both questionnaires with 10 questions were applied to the same set of 114 carefully selected patients with no missing values to questions. An in-depth statistical and reliability analysis was performed on the outcomes provided by Q1, applying a scale and subscale reliability analysis, using the Cronbach’s alpha reliability indicator (Cα). An exploratory analysis called Principal Axis Factoring (PAF) with Oblimin with Kaiser Normalization rotation was applied to prove H1, verifying the assumptions regarding the average variance extracted (AVE) and convergent and discriminant validity (CDV). A scale reliability analysis of outcomes provided by Q2 was performed for proving H2, by calculating Cα. Additionally, a nonparametric correlation analysis was performed, by calculating the Spearman r correlation coefficient between the Rosenberg index and DLQI index, and the 95% confidence interval (CI).

**Results:**

Based on the provided data, the value of Cα obtained in Q1 was 0.84. As a result of applying PAF on Q1, H1 has been proven and two latent factors of self-esteem have been extracted. These factors were named competence (eigenvalue = 4.126; 41.258% of total variance explained) and value (eigenvalue = 1.857; 18.57% of total variance explained). For the two subscales determined by the two factors, we have obtained the Cα values of 0.848 and 0.8, all indicating good reliability. For testing H2, on Q2 data we obtained Cα = 0.914. The Spearman correlation coefficient *r* = −0.734 (*p* < 0.0001), between the self-esteem questionnaire evaluation result and the life-quality index result indicated the existence of a strong negative correlation, which is significant according to 95% CI [−0.81, −0.63].

**Discussion:**

The study focused on analyzing the self-esteem of patients with vitiligo. In addition, the psychometric properties of the Q1 questionnaire were analyzed and Q1 proved to have good internal consistency. PAF indicated a two-factorial structure, with factors called competence and value, proving H1, with a moderate correlation of 0.427 between the two latent constructs. The competence factor includes motivation, self-efficacy, initiative, and persistence in action. The value factor is much more complex, indicating a feeling, a personal evaluation, or a positive or negative attitude toward one’s person, which better captures the entire phenomenology of self-esteem. The statistical analysis of the results provided by the self-esteem questionnaire included questions that proved to be internally consistent. The AVE and CDV assumptions were met. Q2 was proved to have excellent scale reliability. H2 proved a statistically significant strong negative correlation between the Rosenberg score and DLQI score.

## 1 Introduction

Research proved that many dermatology-related illnesses influence the quality of life. Szramka-Pawlak et al. (2014a,b) studied the quality of life and optimism in patients with morphea. Samela et al. (2022) studied the quality of life in patients with non-melanoma skin cancer.

The factors that contribute to the burden severity of the vitiligo disease are multiple, among which the most important are: quality of life, self-esteem, stress, and stigma. Quality of life, in the context of health and illness, is a multidimensional concept that addresses well-being and satisfaction with how health is affected, including emotional state, physical functioning, and social well-being (Simons et al., 2020). These aspects affect the patient’s judgment of how the disease affects his/her life as well as the impact of the disease on his/her professional and personal goals (Divyesh, 2017; Elbuluk and Ezzedine, 2017). Dermatological diseases have a negative impact on the patient’s quality of life due to the symptoms and the condition they induce. Dermatological pathology and, by implication, vitiligo negatively affect school, work, and interpersonal relationships. The treatment itself has a negative effect because of its smell and its greasy vicious appearance (Ongenae et al., 2005; Baranwal et al., 2016). The impact of this disease on the patient’s quality of life has been compared with serious pathologies, such as malignant diseases, hypertension, or diabetes. Although the disease does not produce a direct physical impairment, it can considerably influence the psychological well-being of patients. Unfortunately, vitiligo can cause its patients to feel stigmatized and they may experience increased mood disorders (Ongenae et al., 2006). Parsad et al. reviewed the significant impact that vitiligo can have on the quality of life and highlighted the unnecessary attention vitiligo patients experience while in public (Parsad et al., 2003). In addition to the profound sense of stigma, vitiligo patients also report antagonism, ostracism, and negative comments toward them in public settings (Sarkar et al., 2018). In addition, vitiligo can diminish the patient’s self-image, and these negative feelings are reported to worsen over the years of living with this disease (Silverberg and Silverberg, 2015). Even more, Parsad et al. have suggested that patients who are diagnosed with vitiligo during childhood have an increased risk of psychiatric illness and lifelong effects on self-esteem compared to those diagnosed in adulthood. Furthermore, Bonotis et al. investigated the vitiligo-related quality of life and psychological distress of vitiligo patients using the Dermatological Life Quality Index (DLQI) (Bonotis et al., 2016). The results indicated that patients with a high DLQI, or a greater extent to which vitiligo affects their quality of life, were more likely to express personality traits such as neuroticism. Neuroticism is one of the five higher order personality traits in the psychology study, and this finding highlights that people with vitiligo are more likely to be anxious, angry, worried, envious, depressed, or lonely. Depression, stress, stigma, and mood disorders that can be associated with vitiligo can have a profound impact on a patient’s life (Silvan, 2004; Osinubi et al., 2018). In the study by Passeron (2021) a survey was performed on diverse therapeutics applied to vitiligo in the last 30 years. Ning et al. (2022) studied the relationship between depression and social support among patients with vitiligo in China.

The quality of life of persons with vitiligo has been previously studied in scientific literature. Self-esteem is also an important factor that influences how people live their lives. Based on the comprehensive study of the scientific literature, we identified that the self-esteem of individuals who suffer from vitiligo has not been adequately addressed, despite its significant importance warranting further study. We deemed the research performed by Bibeau et al. (2022) representative for our study, which explores the prevalence and quality of life among adults with vitiligo from Europe, Japan, and the USA. The performed research falls short of addressing the topic of self-esteem among individuals with vitiligo. Furthermore, the correlation between self-esteem and quality of life among individuals with vitiligo has not been sufficiently studied.

## 2 Materials and methods

### 2.1 Study design, survey, and participants

Patients diagnosed with vitiligo from outpatient offices and the outpatient department of the Dermatology Clinic in Târgu Mureș participated in the study, adhering to the specified inclusion and exclusion criteria. The study period was between March 2021 and March 2022. Inclusion criteria encompassed patients over 18 years of age, diagnosed with vitiligo of any form, and who signed the informed consent. Exclusion criteria included patients under 18 years of age, diagnosed with vitiligo of any form, and those who did not sign the informed consent. Cases with missing responses to one or more questions were also excluded. To carry out the study, we obtained the approval of the Ethics Commission of the Faculty of Medicine with no. 1255/2021 and the Mureș County Clinical Hospital with no. 16501/2021. Patients completed two questionnaires: one on self-esteem called the Rosenberg questionnaire (Q1) (Rosenberg, 1965) and the other on the quality of life called DLQI (Q2) (Finlay and Khan, 1994). These questionnaires contain 10 questions each with 4 or 5 answer options. The questionnaires were completed using paper and pencil, with no time limit. At the same time, a complete dermatological clinical examination was performed by experienced dermatologists involved, voluntarily. All patients whose data were included in the research signed the informed consent. The data collected from the questionnaires and the clinical examination were recorded in a database. The data has been encoded in compliance with the General Data Protection Regulation (GDPR).

### 2.2 Statistical analyses

Internal consistency measures how well a set of items/variables/questions are grouped. We have measured the internal consistency using the Cronbach alpha (Cα) reliability indicator. Cho (2016) presents the interpretation of the internal consistency that we approached.

For Q1, an in-depth statistical scale and subscale Cα reliability analysis was performed on the questionnaire outcomes. For Q1, the results of descriptive statistics included the mean, standard deviation (SD), and other indicators. For the verification of data normality assumption of the variables from Q1, the Lilliefors goodness-of-fit (Lill) (Dallal and Wilkinson, 1986) test was applied at the 0.05 significance level. For additional visual validation of the normality, a representation using a Q-Q plot (Wilk and Gnanadesikan, 1968) for each variable was performed (However, these visual Q-Q plot representations were not included in this manuscript). For the verification of how each variable influences the internal consistency (if the questions give results that are consistent mathematically with each other), the value of Cα was calculated for each of the situations where each variable was removed in succession. We analyzed the correlations of all the variables with each other. The correlation coefficient r was calculated according to the appropriateness (Stigler, 1989; Bonett and Wright, 2000) of the application of parametric and nonparametric calculus. We have analyzed the significance at both 2-tailed significance levels 0.01 and 0.05. For each case/patient, ResultSelfEsteem is calculated, which presents its evaluated self-esteem. This can be referred as Rosenberg’s index. ResultSelfEsteem is the variable that summarizes the final self-esteem test results, on which we have performed descriptive statistics and normality analysis, finally calculating its correlation with all the variables that constitute Q1. The ResultSelfEsteem is calculated based on the indications in the questionnaire, initially for some variables making reverse coding. Subsequently, the values in each row of self-esteem are calculated by adding the values of corresponding questions within that row.

Hauben et al. (2017) present the interpretation of the Kaiser-Meyer-Olkin measure of sampling adequacy (KMO) test results that we adopted.

Exploratory factor analysis (EFA) is used to determine the underlying latent factor structure of a set of variables (Prather et al., 1997). Based on the principle that a causal model exists, we have performed a so-called Principal axis factoring (PAF) (Grieder and Steiner, 2022). For rotation, we selected the Oblimin with Kaiser Normalization method verifying that the extracted latent factors are correlated. The determinant of the correlation matrix of all the variables can be regarded as an indicator of multicollinearity. We have verified all the assumptions necessary to be passed for the correct application of the PAF, including the value of the determinant of a correlation matrix, which should be higher than 0.00001 (Field, 2000); KMO test assumption, where KMO ≥ 0.6; and Bartlett’s Test of Sphericity (BTS) (Hauben et al., 2017) test assumption, corresponding to the *p*-value of BTS, where *p-*value < 0.05. We have calculated the initial and extracted communalities (McDonald, 1985).

Determining the appropriate number of factors is one of the most difficult decisions (Choi et al., 2017; Iantovics et al., 2019) to take in PAF and Exploratory Factor Analysis (EFA) in general. For the precise establishment of the number of factors, we considered the decision rule based on the following combination of criteria: (A) the Kaiser rule, which suggests selecting only the factors with eigenvalues greater than 1 (Kaiser, 1960); (B) identifying the inflation point on the Scree plot visual interpretation (Cattell, 1966), and (C) adhering to the criterion regarding the total cumulative variances explained, as indicated in the research (Iantovics et al., 2019).

The pattern matrix (Ayr et al., 2009) presents the loadings of the variables into the factors after rotation. Additionally, for a visual representation, we have plotted the factor plot in rotated factor space (Hauben et al., 2017).

Additionally, for the correctness verification of the decision for choosing the varimax orthogonal rotation, we have analyzed the correlation between the two extracted factors F1 and F2 by obtaining the factor correlation matrix. For an orthogonal rotation to be applicable, the average value of the correlation coefficients between all the factors must be lower than or equal to 0.3.

The assumptions regarding the average variance extracted (AVE) (Nia et al., 2020), convergent validity (Nia et al., 2020), and discriminant validity (CDV) (Nia et al., 2020) were verified.

To prove H2, a reliability analysis of Q2 was performed, and we made a correlation analysis, by calculating the Spearman r nonparametric correlation coefficient between the Rosenberg Index and DLQI Index, and the 95% CI of r.

## 3 Results

For increased readability, we present the obtained results and discuss them treating the correctness of the statistical tests applied.

### 3.1 Participants’ characteristics

Richard et al. (2022) presented a population-based study of the prevalence of the most common skin diseases in Europe. Vitiligo is a rare condition, affecting between 0.5 and 4% of the global population (Krüger and Schallreuter, 2012; Al-Harbi, 2013). The highest incidence has been reported in India, followed by Mexico and Japan (Sehgal and Srivastava, 2007).

Our study consisted of 114 patients, all of whom were Caucasians with Fitzpatrick skin types ranging from I-III. Out of the total patients, 56 (49.12%, 95%CI 39.81–58.44%) patients were male and 58 (50.88%, 95% CI 41.56–60.19%) patients were female. The average age was 49, with 95%CI [45.8, 52.3], median age 49, range 59, with minimum 19 and maximum 78 years. Of the patients, 76 (66.67%) of them reside in urban areas, while 38 (33.33%) are from rural areas. In terms of marital status, 77 (67.54%) were married and 37 (32.46%) patients were unmarried.

### 3.2 Descriptive statistics and data reliability analysis of Q1 outcome

Experimental setup of Q1: number of items/questions/variables = 10, denoted as RQ = {RQ1, RQ2, RQ3, RQ4, RQ5, RQ6, RQ7, RQ8, RQ9, RQ10}; number of valid cases, *n* = 114. Cα = 0.84 calculated with all variables included indicates a good internal consistency, 0.84 ∈[0.8, 0.9].

Table 1 presents the descriptive statistics of the RQ variables.

**Table 1:** Descriptive statistics of the RQ variables.

Variable	Mean	SD
RQ1	1.85	1.075
RQ2	1.89	0.954
RQ3	1.18	1.110
RQ4	1.85	0.895
RQ5	1.33	1.126
RQ6	1.49	1.033
RQ7	1.45	1.040
RQ8	0.84	0.992
RQ9	0.99	1.077
RQ10	0.82	1.085

RQ1, RQ2, … RQ10 variables’ normality was verified using the Lill test (Table 2). None of the studied variables RQ1, RQ2, … RQ10 met the normality assumption even at the 0.001 significance level (*p-*value < 0.001). The result of visual additional validation of the normality of each variable using a Q-Q plot was the same as the numerical evaluation.

**Table 2:** Lilliefors test results.

Variable	Statistic	df	*p*-value
RQ1	0.217	114	0
RQ2	0.201	114	0
RQ3	0.225	114	0
RQ4	0.259	114	0
RQ5	0.198	114	0
RQ6	0.198	114	0
RQ7	0.211	114	0
RQ8	0.293	114	0
RQ9	0.278	114	0
RQ10	0.335	114	0

For verification, if there are questions with responses mathematically inconsistent with others, we calculated the value of Cα for each variable when deleted (Table 3). The results presented in the column labeled “Cronbach’s alpha if variable deleted” present the changes in the value of the Cα when a single specific variable is deleted. The differences of these values ranging from 0.84 indicates the amount of change in the reliability in case of variable deletion. The results prove that deleting variables does not significantly change the value of reliability.

**Table 3:** Cronbach’s alpha if variable deleted, with initial Cα = 0.84 for all the variables included.

Variable that considered deleted	Cronbach’s alpha if variable deleted	Cα - Cronbach’s alpha if variable deleted
RQ1	0.822	0.018
RQ2	0.830	0.01
RQ3	0.827	0.013
RQ4	0.832	0.008
RQ5	0.827	0.013
RQ6	0.835	0.005
RQ7	0.823	0.017
RQ8	0.818	0.022
RQ9	0.821	0.019
RQ10	0.812	0.028

Table 4 presents the correlation coefficients between all the variables RQ. Since the variables do not meet the normality assumption, we have chosen to calculate the nonparametric Spearman r correlation coefficient. The significant correlations at the 0.01 and 0.05 significance levels are marked in the table with ** correlation significance at the 0.01 level, and * correlation significant at the 0.05 level.

**Table 4:** Correlations between the RQ variables.

		RQ1	RQ2	RQ3	RQ4	RQ5	RQ6	RQ7	RQ8	RQ9	RQ10
RQ1	*r*	–	0.621^**^	0.241^**^	0.392^**^	0.265^**^	0.473^**^	0.502^**^	0.337^**^	0.194^*^	0.342^**^
	Sig.		0	0.010	0	0.004	0	0	0	0.038	0
RQ2	*r*	0.621^**^	–	0.074	0.407^**^	0.195^*^	0.374^**^	0.609^**^	0.232^*^	0.078	0.198^*^
	Sig.	0		0.434	0	0.038	0	0	0.013	0.411	0.035
RQ3	*r*	0.241^**^	0.074	–	0.118	0.546^**^	0.07	0.137	0.526^**^	0.5^**^	0.554^**^
	Sig.	0.010	0.434		0.213	0	0.461	0.146	0	0	0
RQ4	*r*	0.392^**^	0.407^**^	0.118	–	0.347^**^	0.335^**^	0.465^**^	0.246^**^	0.179	0.194^*^
	Sig.	0	0	0.213		0	0	0	0.008	0.057	0.038
RQ5	*r*	0.265^**^	0.195^*^	0.546^**^	0.347^**^	–	0.228^*^	0.220^*^	0.391^**^	0.378^**^	0.351^**^
	Sig.	0.004	0.038	0	0		0.015	0.018	0	0	0
RQ6	*r*	0.473^**^	0.374^**^	0.07	0.335^**^	0.228^*^	–	0.413^**^	0.182	0.242^**^	0.198^*^
	Sig.	0	0	0.461	0	0.015		0	0.052	0.010	0.035
RQ7	*r*	0.502^**^	0.609^**^	0.137	0.465^**^	0.220^*^	0.413^**^	–	0.349^**^	0.261^**^	0.224^*^
	Sig.	0	0	0.146	0	0.018	0		0	0.005	0.016
RQ8	*r*	0.337^**^	0.232^*^	0.526^**^	0.246^**^	0.391^**^	0.182	0.349^**^	–	0.588^**^	0.63^**^
	Sig.	0	0.013	0	0.008	0	0.052	0		0	0
RQ9	*r*	0.194^*^	0.078	0.5^**^	0.179	0.378^**^	0.242^**^	0.261^**^	0.588^**^	–	0.707^**^
	Sig.	0.038	0.411	0	0.057	0	0.01	0.005	0		0
RQ10	*r*	0.342^**^	0.198^*^	0.554^**^	0.194^*^	0.351^**^	0.198^*^	0.224^*^	0.63^**^	0.707^**^	–
	Sig.	0	0.035	0	0.038	0	0.035	0.016	0	0	

**Correlation is significant at the 0.01 level (2-tailed). *Correlation is significant at the 0.05 level (2-tailed).

Table 5 presents the descriptive statistics of ResultSelfEsteem (Rosenberg’s Index).

**Table 5:** Descriptive statistics of Rosenberg’s Index (ResultSelfEsteem variable).

Indicator	Statistic	Standard error
Mean	13.7	0.623
95% confidence interval for mean [lower bound, upper bound]	[12.47, 14.94]	
5% trimmed mean	13.48	
Median	13	
Variance	44.317	
Standard deviation	6.657	
Minimum	3	
Maximum	30	
Range	27	
Interquartile range	9	
Skewness	0.467	0.226
Kurtosis	−0.418	0.449

**Figure 1:**
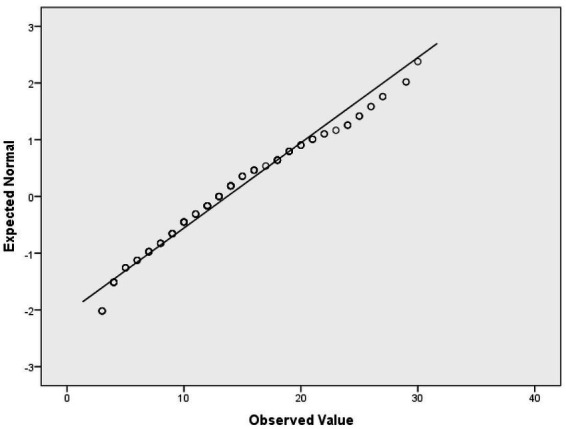
Q-Q plot of ResultSelfEsteem.

For verification, if the ResultSelfEsteem variable is normally distributed, we have applied the Lill test at a 0.05 significance level (Table 6). We have also applied the Shapiro–Wilk (SW) test (Shapiro and Wilk, 1965) at the 0.05 significance level (Table 6) since the SW test power is higher (Razali and Wah, 2011) than the test power of the Kolmogorov Smirnov (Lilliefors, 1969; Morar et al., 2018), Anderson Darling, and Lill tests. Both Lill and SW test results indicate that ResultSelfEsteem fails to meet the normality assumption. Additionally, the Q-Q plot of ResultSelfEsteem (Figure 1) visually validated the result of numerical goodness-of-fit normality test results.

**Table 6:** Normality test of the Rosenberg’s Index (ResultSelfEsteem variable).

Lilliefors			Shapiro–Wilk		
Statistic	df	*p*-value	Statistic	df	*p*-value
0.105	114	0.004	0.966	114	0.005

Table 7 presents the strength of correlation of the variable ResultSelfEsteem with each of the variables RQ1, RQ2, … RQ10. Since each variable failed to meet the normality assumption, we have calculated the Spearman r correlation coefficient, and the corresponding 95% CI to prove that r indicates a statistically significant positive correlation (the lower limit of the confidence interval is higher than 0). According to the obtained results, each of the correlations is significant at the 0.01 significance level (2-tailed).

**Table 7:** Spearman r correlations between RQ1, RQ2, … RQ10 and ResultSelfEsteem.

ResultSelfEsteem		RQ1	RQ2	RQ3	RQ4	RQ5	RQ6	RQ7	RQ8	RQ9	RQ10
	r	0.698	0.6	0.58	0.58	0.62	0.54	0.67	0.68	0.61	0.64
	*p*-value	0.0001	0.0001	0.0001	0.0001	0.0001	0.0001	0.0001	0.0001	0.0001	0.0001
	95%CI	0.587–0.784	0.46–0.71	0.44–0.69	0.44–0.7	0.49–0.73	0.39–0.66	0.55–0.76	0.57–0.77	0.49–0.72	0.52–0.75

**Figure 2:**
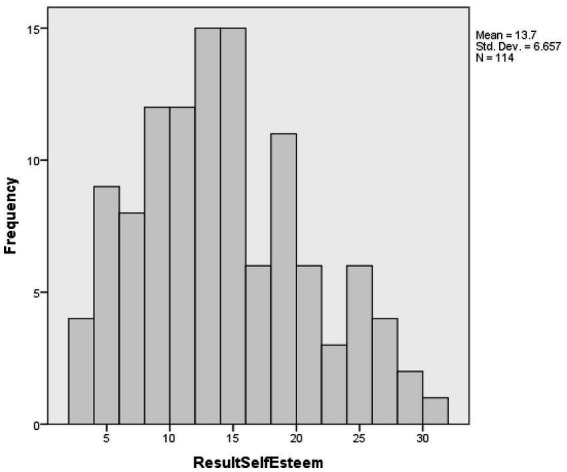
Histogram of self-esteem of vitiligo patients.

Figure 2 presents the histogram of self-esteem results of vitiligo patients.

**Figure 3:**
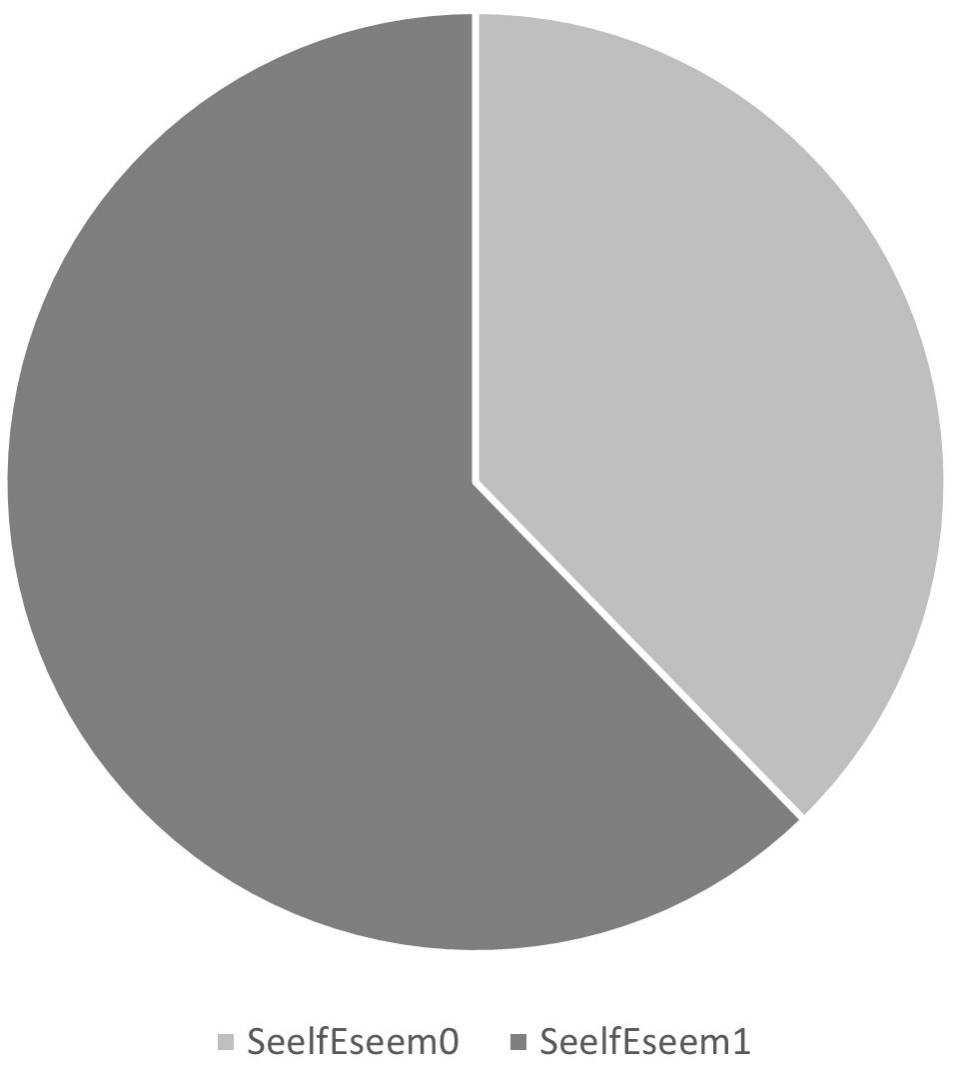
Number of individuals with normal and decreased self-esteem.

We have created a dummy variable named SelfEsteemClass with the SeelfEseem1 and SeelfEseem0 indicators included, according to the Rosenberg indication of interpretation of the questionnaire results. SelfEseem1 is associated with the values 0–14 from ResultSelfEsteem. SeelfEseem0 is associated with the values between 15 and 25 from ResultSelfEsteem. In the case of a person according to the test results interpretation: SeelfEseem1 marks that the self-esteem of a person is problematic; SeelfEseem0 marks that the self-esteem is not problematic for that person. In SelfEsteemClass (Figure 3), the SeelfEseem1 is counted 71 times (62.28%) and SeelfEseem0 is counted 43 times (37.2%), which indicates a relatively balanced set of data of individuals with normal self-esteem and decreased self-esteem.

### 3.3 Principal axis factoring for identifying the latent factors of self-esteem

Initially, all the mandatory assumptions necessary to be met were verified for the PAF application to be correct.

The correlation matrix of all the variables RQ1, … RQ10 was calculated. The determinant of the correlation matrix was found to be 0.015, which met the assumption of being higher than 0.00001. The KMO test assumption was fulfilled, with KMO = 0.807, where KMO ≥ 0.6 is considered meritorious. Additionally, the BTS test assumption was met, with value of *p* = 0, value of *p* < 0.05, approx. Chi-square = 457.431, and df = 45.

Table 8 presents the initial and extracted communalities. All the extracted communalities passed the threshold by 0.2. Based on this fact, it was not necessary to remove any of them.

**Table 8:** Initial and extracted communalities.

Variable	Initial	Extraction
RQ1	0.481	0.526
RQ2	0.510	0.612
RQ3	0.512	0.542
RQ4	0.308	0.317
RQ5	0.399	0.335
RQ6	0.307	0.300
RQ7	0.500	0.561
RQ8	0.488	0.536
RQ9	0.615	0.626
RQ10	0.657	0.701

**Figure 4:**
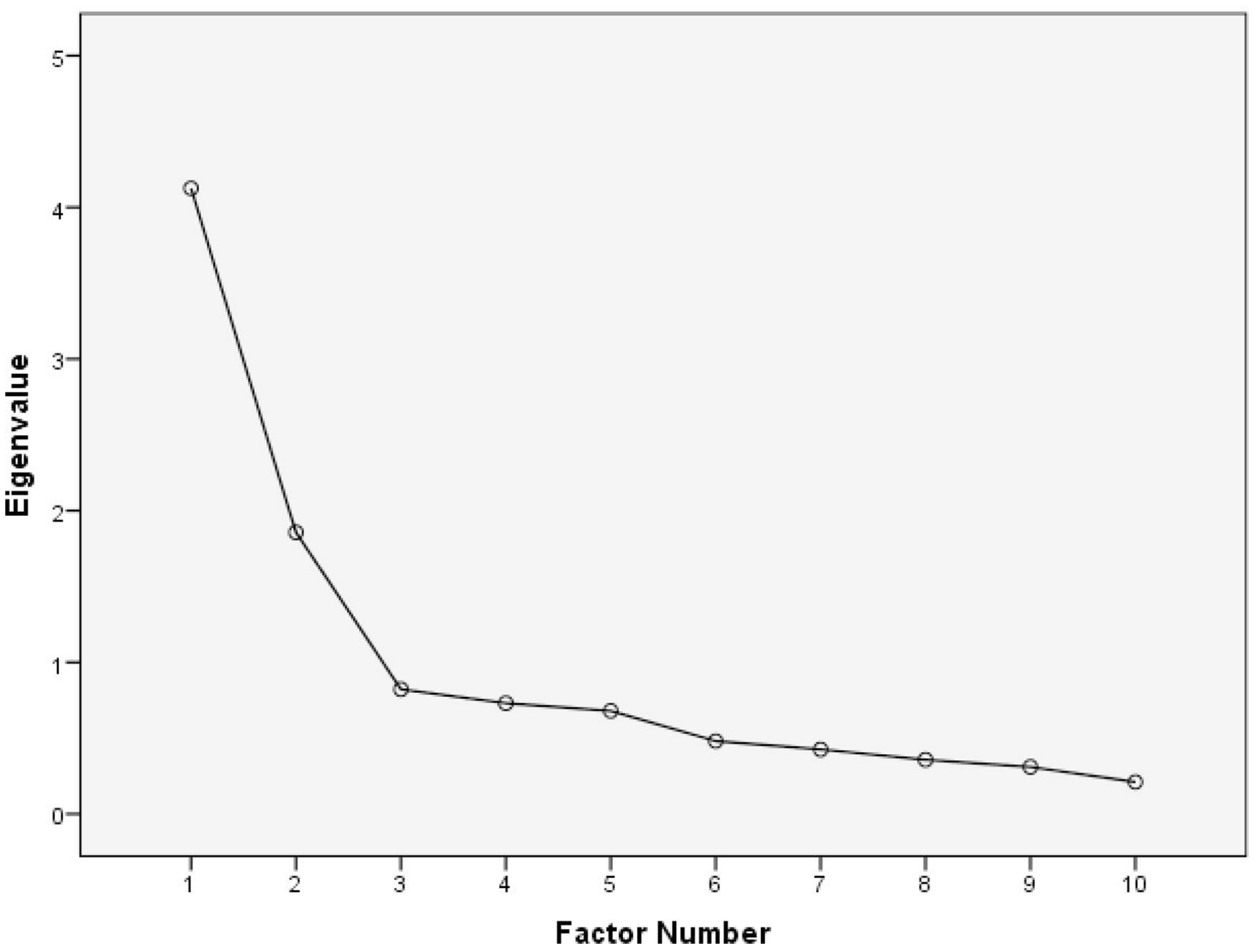
Scree plot for visual estimation of the number of factors.

Table 9 presents the results regarding the extraction of the factors, including the calculated eigenvalues and the variance explained. Figure 4 presents the Scree plot. According to the rules (A), (B), and (C), we concluded the extraction of the two latent factors, denoted as F1 (with the eigenvalue 4.126, 41.258% of the total variance explained) and F2 (with the eigenvalue 1.857, and 18.57% of the total variance explained). The total cumulative variance of these two factors is 59.83% which, according to Iantovics et al. (2019) can be considered appropriate. We refer to the factor F1, competence, and factor F2, value.

**Table 9:** Factor extraction, variance explained.

Factor	Initial eigenvalues			Extraction sums of squared loadings			Rotation sums of squared loadings		
	Total	% of variance	Cumulative %	Total	% of variance	Cumulative %	Total	% of variance	Cumulative %
F1	4.126	41.258	41.258	3.658	36.584	36.584	2.695	26.948	26.948
F2	1.857	18.570	59.829	1.398	13.98	50.564	2.362	23.616	50.564
3	0.822	8.221	68.050						
4	0.731	7.308	75.358						
5	0.68	6.797	82.155						
6	0.481	4.809	86.964						
7	0.425	4.254	91.218						
8	0.357	3.572	94.790						
9	0.31	3.102	97.892						
10	0.211	2.108	100						

**Figure 5:**
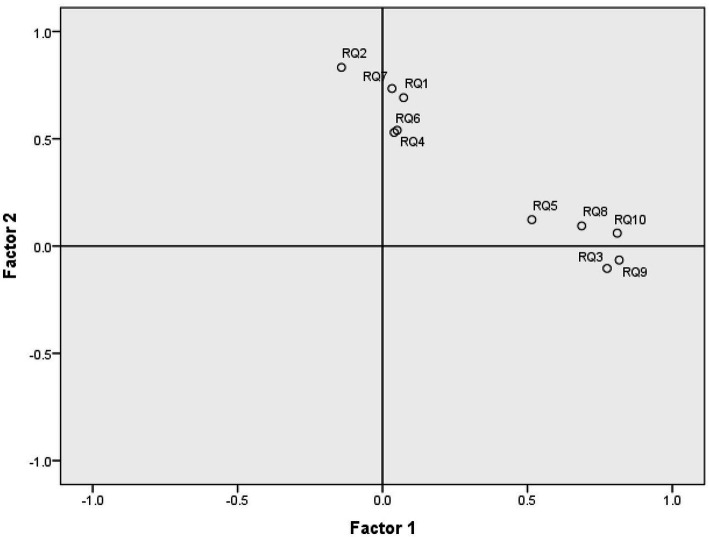
Factor plot in rotated factor space.

Table 10, the pattern matrix, presents the loadings of the variables into the factors after performing the oblique rotation. The factor loadings lower than 0.25 were suppressed based on the consideration that they are not relevant. All the remaining factor loadings passed the threshold by 0.5. RQ9, RQ10, RQ3, RQ8, and RQ5 are loaded into F1, with factor loadings varying between 0.515 and 0.817. We performed a reliability analysis for the subscale RQ9, RQ10, RQ3, RQ8, and RQ5 obtaining Cα = 0.848, which indicates good internal reliability. RQ2, RQ7, RQ1, RQ4, and RQ6, are loaded into F2, with factor loadings varying between 0.530 and 0.832. We have performed a reliability analysis for the subscale RQ2, RQ7, RQ1, RQ4, RQ6, obtaining Cα = 0.8, which indicates good internal reliability. In Table 10, the variables are in descending order based on the value of loadings. Figure 5 presents the factor plot in rotated factor space. Since all the factor loadings are positive, it can be concluded that the numbering/coding of the values that can be chosen in the questions is realized appropriately (reverse coding is not used for any of the variables). The proportion of variance accounted for by the two factors in combination according to Table 9 is 50.56%.

**Table 10:** Pattern matrix.

Variables	Factor	
	F1 (competence)	F2 (value)
RQ9	0.816635	
RQ10	0.809992	
RQ3	0.774852	
RQ8	0.687085	
RQ5	0.515249	
RQ2		0.832442
RQ7		0.734151
RQ1		0.691506
RQ4		0.539625
RQ6		0.529552

Table 11 presents the factor correlation matrix between F1 and F2. The correlation coefficient between F1 and F2 is 0.427, which indicates that the choice of the oblique type of rotation, in our case the Oblimin with Kaiser Normalization, is appropriate. It corresponds to the decision rule that the average correlation between all the factors must be higher than 0.3.

**Table 11:** Factor correlation matrix.

Factor	F1	F2
F1	–	0.427
F2	0.427	–

Using results obtained in Table 10, the AVE is calculated, and CDV verification is performed. The average loading of F1 was 0.721 (0.7207626), which passed the threshold by 0.6. The average loading of F2 was 0.665 (0.6654552), which passed the threshold by 0.6. AVE by F1 was 0.52 (0.519498726), which passed the threshold by 0.4. AVE by F2 was 0.43 (0.442830623), which passed the threshold by 0.40. The obtained results prove that the convergent validity assumptions of the two factors were met. The variance extracted between F1 and F2 was 0.48 (0.481164674). The correlation *r* between F1 and F2 was by 0.427, with *r*^2^ = 0.18 (0.182329). Since variance extracted between F1 and F2 > *r*^2^ (0.481164674 > 0.182329), the discriminant validity is also passed.

## 4 Discussion

We have performed a Principal Axis Factoring to identify the latent factors of self-esteem. This section presents the correlation analysis between the self-esteem and Life-Quality Index.

The ResultSelfEsteem variable contains the Rosenberg score of the self-esteem questionnaire. The increase in Rosenberg’s score represents an increase in self-esteem. A Rosenberg score value that is lower than 15 is an indicator that the self-esteem of the vitiligo patient is an issue.

ResultLifeQuality variable represents the DLQI score that summarizes the life quality questionnaire results. For each case/patient, the ResultLifeQuality is calculated, which presents its evaluated Life Quality Index. ResultLifeQuality is calculated according to the questionnaire indications, after which the values in each row (case) are calculated by adding the values for each question from the same row. The increase in the DLQI score indicates an increase in the effect of vitiligo on the quality of life. For the Q2 questionnaire data, 114 patients obtained Cα = 0.955 0.914, indicating excellent reliability.

We have analyzed the hypothesis of the correlation between the Rosenberg score and the DLQI score of patients who suffer from vitiligo. Since both variables failed to meet the normality assumption, we have chosen the calculus of the nonparametric Spearman r correlation coefficient, obtaining *r* = −0.73 (*p* < 0.0001), with a 95% CI [−0.81, −0.63]. Since *r* < 0 and −0.63 < 0, the existence of a significant negative correlation is proved. However, the obtained result proves H2 which claims that the increase in self-esteem is an indicator of the decrease in the DLQI score. Based on the literature research, we can conclude that our article is the only one that analyzes the correlation between self-esteem and Life Quality Index, in the case of vitiligo patients.

## 5 Conclusion

In this study, the psychometric properties of the Rosenberg self-esteem questionnaire (Q1) for vitiligo patients were assessed. Q1 proved to have good internal consistency. PAF indicated a two-factorial structure, which was identified as being named competence and value, proving H1, with a moderate correlation of 0.427 between the two latent constructs. The competence factor includes motivation, self-efficacy, initiative, and persistence in action. The value factor is much more complex, indicating a feeling, a personal evaluation, or a positive or negative attitude toward one’s person, which better captures the entire phenomenology of self-esteem. The statistical analysis of the results provided by the self-esteem questionnaire included questions that proved to be internally consistent. DLQI questionnaire (Q2) was proved to have excellent scale reliability. With H2 proven, a statistically significant strong negative correlation was obtained between the Rosenberg and DLQI scores.

Subsequently, this study can be considered as a guide for the correct application of the EAF, which involves many assumptions that are not frequently verified by researchers. Additional information regarding performed statistical analyses are presented in the Supplementary material. EAF also involves some decisions, such as those regarding the establishment of the number of factors to be extracted that are challenging (Choi et al., 2017; Iantovics et al., 2019). As a decision rule, for the establishment of the correct number of extracted factors, we presented a combination of criteria that included the Kaiser criterion, Scree plot visual interpretation, and total variance explained, followed by validation using parallel analysis involving Monte Carlo simulation.

## Data availability statement

The raw data supporting the conclusions of this article will be made available by the authors, without undue reservation.

## Ethics statement

The studies involving humans were approved by Ethics Commission of the Faculty of Medicine with no. 1255/2021, respectively of the Mureș County Clinical Hospital with no. 16501/2021. The studies were conducted in accordance with the local legislation and institutional requirements. The participants provided their written informed consent to participate in this study.

## Author contributions

LF, LI, and GF conceptualized the study and developed the methodology, validated the results and carried out formal analysis, and were responsible for visualization. LI was responsible for the software used in the study, curated the data, and wrote the original draft, while LF, LI, and GF reviewed and edited it. LF and GF conducted the investigation and provided resources for the study. GF supervised the study and managed the project, while also acquiring the necessary funding. All authors contributed to the article and approved the submitted version.
