# Supplementary material for: Exploratory axis factoring for identifying the self-esteem latent factors and their correlation with the life quality of persons suffering from vitiligo
Source: Front Psychol. 2023 Dec 15;14:1200713. doi: 10.3389/fpsyg.2023.1200713 (PMC10757324; doi:10.3389/fpsyg.2023.1200713)
Supplement: Supplementary file 1 [file Data_Sheet_1.pdf]

Supplementary material

Our secondary aim was to present the performed statistical modeling as a guide that can be followed by other researchers. In this sense, we performed a comprehensive statistical analysis. To increase the readability of the main manuscript in this supplementary manuscript, we have included some statistical modeling details.

A1. Additional details to the performed statistical analyses

This section presents some additional details regarding Section 2.2 entitled “Statistical analyses” from the main manuscript.

For rigorous verification, if there are candidate variables for removal, we have analyzed the anti-image matrices (Hauben et al., 2017). The values from the matrix diagonal measure the Sampling Adequacy (MSA) indicating how well each variable is correlated with all others. Each of the values from the diagonal must pass the threshold by 0.6, otherwise, it is an indication of the fact that the corresponding variable to the value can be removed.

We have calculated the initial and extracted communalities (McDonald, 1985). Extracted communalities are estimates of the variance in each variable accounted for by the factors in the factor solution. The extracted communality is specific to each variable, it represents the common variance explained in each variable by the factors. As a threshold for removing a variable, we consider the extracted communality that has a value lower than or equal to 0.25.

The application of the decision rule regarding the number of factors to extract was followed by a validation using Parallel Analysis based on a Monte Carlo method (Horn, 1965).

The Reproduced Correlations (Hauben et al., 2017) and the Corresponding Residuals (Hauben et al., 2017) were calculated to verify the designed model.

A2. Additional details to PAF for identifying the latent factors of self-esteem

In this section, there are presented some additional details regarding Section 3.3 entitled “PAF for identifying the latent factors of self-esteem” from the manuscript.

Table A1 presents the results of the anti-image correlation matrices. Each of the values from the diagonal passed the threshold by 0.6, which indicates that none of the variables must be removed.

[Insert Table A1 here]

For validation of the correctness of the number of extracted factors, we have performed a Parallel Analysis (Horn, 1965) based on Monte Carlo simulation (Table A2), with 10 variables, 114 subjects, and 1000 replications. The columns labeled “Eigenvalue number”, “Random Eigenvalue ", “Standard Deviation” present the results of the simulation. The column labeled “Prospective factor number” indicates the prospective number of the factor, which, if the decision passes, is indicated to be selected. The column labeled “Initial eigenvalues" represents the initial eigenvalues that are shown in Table 9 (manuscript). The last column, labeled “Decision rule”, presents the result of the decision rule applied for the selection of a factor with a certain number. According to the decision rule if “Initial

eigenvalue”>” Random Eigenvalue” then the corresponding factor to the eigenvalue number must be selected. However, the number of selected factors using the decision rule is validated.

[Insert Table A2 here]

Table A3 presents the Reproduced Correlations, where *indicates the reproduced communalities.

[Insert Table A3 here]

Table A4 represents the residuals that are computed between observed and reproduced correlations. The obtained results indicate that the rule regarding the % of residuals with absolute values greater than 0.05 is less than 50% is met.

[Insert Table A4 here]

References

Hauben, M., Hung, E., and Hsieh, W.Y. (2017). An exploratory factor analysis of the spontaneous reporting of severe cutaneous adverse reactions. *Therapeutic Advances in Drug Safety*, 8(1), 4-16.

Horn, J.L. (1965). A rationale and test for the number of factors in factor analysis. *Psychometrika*, 30, 179-185.

McDonald, R.P. (1985). Factor analysis and related methods. Hillside, NJ: Lawrence Erlbaum Associates, Inc.

List of tables.

Table A1 Anti-image correlation matrices

	**RQ1**	**RQ2**	**RQ3**	**RQ4**	**RQ5**	**RQ6**	**RQ7**	**RQ8**	**RQ9**	**RQ10**
**RQ1**	0.826[a]	-0.352	-0.114	-0.060	0.010	-0.284	-0.092	-0.083	0.151	-0.155
**RQ2**	-0.352	0.754[a]	0.063	-0.087	-0.038	-0.023	-0.420	0.043	0.190	-0.161
**RQ3**	-0.114	0.063	0.801[a]	0.114	-0.432	0.152	0.002	-0.178	-0.104	-0.211
**RQ4**	-0.06	-0.087	0.114	0.853[a]	-0.247	-0.086	-0.237	-0.015	0.021	-0.044
**RQ5**	0.01	-0.038	-0.432	-0.247	0.801[a]	-0.116	0.053	-0.049	-0.062	0.023
**RQ6**	-0.284	-0.023	0.152	-0.086	-0.116	0.825[a]	-0.137	0.085	-0.157	-0.015
**RQ7**	-0.092	-0.42	0.002	-0.237	0.053	-0.137	0.794[a]	-0.186	-0.177	0.145
**RQ8**	-0.083	0.043	-0.178	-0.015	-0.049	0.085	-0.186	0.898[a]	-0.174	-0.241
**RQ9**	0.151	0.19	-0.104	0.021	-0.062	-0.157	-0.177	-0.174	0.764[a]	-0.554
**RQ10**	-0.155	-0.161	-0.211	-0.044	0.023	-0.015	0.145	-0.241	-0.554	0.790[a]

[a] MSA

Table A2 Results of Parallel Analysis using Monte Carlo simulation

**Prospective factor number**	**Random Eigenvalue**	**Standard Deviation**	**Initial eigenvalue**	**Result of decision rule**
1	1.494	0.085	4.126	**Passed**
2	1.333	0.057	1.857	**Passed**
3	1.213	0.048	0.822	Not Passed
4	1.118	0.042	0.731	Not Passed
5	1.025	0.037	0.68	Not Passed
6	0.933	0.041	0.481	Not Passed
7	0.85	0.04	0.425	Not Passed
8	0.77	0.042	0.357	Not Passed
9	0.69	0.048	0.31	Not Passed
10	0.58	0.049	0.211	Not Passed

Table A3 Reproduced Correlations

	RQ1	RQ2	RQ3	RQ4	RQ5	RQ6	RQ7	RQ8	RQ9	RQ10
**RQ1**	0.526[a]	0.549	0.210	0.408	0.278	0.397	0.542	0.321	0.253	0.341
**RQ2**	0.549	0.612[a]	0.085	0.427	0.205	0.417	0.574	0.219	0.124	0.22
**RQ3**	0.210	0.085	0.542[a]	0.159	0.404	0.149	0.19	0.523	0.582	0.605
**RQ4**	0.408	0.427	0.159	0.317[a]	0.214	0.308	0.421	0.246	0.193	0.261
**RQ5**	0.278	0.205	0.404	0.214	0.335[a]	0.204	0.27	0.422	0.441	0.481
**RQ6**	0.397	0.417	0.149	0.308	0.204	0.3[a]	0.41	0.234	0.182	0.249
**RQ7**	0.542	0.574	0.190	0.421	0.270	0.41	0.561[a]	0.308	0.234	0.325
**RQ8**	0.321	0.219	0.523	0.246	0.422	0.234	0.308	0.536[a]	0.569	0.612
**RQ9**	0.253	0.124	0.582	0.193	0.441	0.182	0.234	0.569	0.626[a]	0.656
**RQ10**	0.341	0.22	0.605	0.261	0.481	0.249	0.325	0.612	0.656	0.701[a]

a reproduced communalities

Table A4 Residuals corresponding to the reproduced correlations

Variable	RQ1	RQ2	RQ3	RQ4	RQ5	RQ6	RQ7	RQ8	RQ9	RQ10
**RQ1**		0.04	0.044	-0.045	-0.017	0.060	-0.055	0.006	-0.055	0.029
**RQ2**	0.04		0.017	-0.031	-0.015	-0.059	0.028	-0.003	-0.039	0.035
**RQ3**	0.044	0.017		-0.033	0.155	-0.067	-0.017	0.002	-0.062	-0.033
**RQ4**	-0.045	-0.031	-0.033		0.117	0.021	0.041	-0.013	-0.01	-0.025
**RQ5**	-0.017	-0.015	0.155	0.117		0.027	-0.044	-0.034	-0.052	-0.075
**RQ6**	0.06	-0.059	-0.067	0.021	0.027		-0.007	-0.054	0.069	0.007
**RQ7**	-0.055	0.028	-0.017	0.041	-0.044	-0.007		0.053	0.046	-0.04
**RQ8**	0.006	-0.003	0.002	-0.013	-0.034	-0.054	0.053		0.01	0.01
**RQ9**	-0.055	-0.039	-0.062	-0.010	-0.052	0.069	0.046	0.01		0.077
**RQ10**	0.029	0.035	-0.033	-0.025	-0.075	0.007	-0.04	0.01	0.077	
